# Microbial Resistance in Urinary Tract Infections

**DOI:** 10.7759/cureus.8110

**Published:** 2020-05-14

**Authors:** Jahanzeb Malik, Nismat Javed, Farhan Malik, Uzma Ishaq, Zubair Ahmed

**Affiliations:** 1 Cardiology, Rawalpindi Institute of Cardiology, Rawalpindi, PAK; 2 Internal Medicine, Shifa College of Medicine - Shifa Tameer-E-Millat University, Islamabad, PAK; 3 Internal Medicine, Blackpool Teaching Hospitals, NHS Foundation Trust, Blackpool, England, GBR; 4 Hematology and Medical Oncology, Fauji Foundation Hospital, Rawalpindi, PAK; 5 Internal Medicine, Benazir Bhutto Hospital Rawalpindi, Rawalpindi, PAK

**Keywords:** uti, pakistan, microbes, resistance

## Abstract

Objective

To determine the pattern of microbes responsible for urinary tract infections and their susceptibility to antimicrobial agents.

Methods

This was a prospective, observational study conducted at Benazir Bhutto Hospital, Rawalpindi, Pakistan. The urine samples of 440 patients were collected and sent for culture and sensitivity analysis. The results were recorded on a proforma. The data were analyzed using IBM Statistical Package for Social Sciences (SPSS) version 22 (IBM Corp., Armonk, NY). Descriptive statistics were used to describe the data. Chi-square test was applied to determine the significance of the difference between gender and microorganisms as well as microorganism and antimicrobial sensitivity. P-value of less than 0.05 was considered significant.

Results

Out of 440 urine samples, 144 culture-positive samples had been obtained from male participants and 296 culture-positive samples had been obtained from female participants. The most common organism on analysis was Escherichia coli. There were more rates of resistance in males. The organisms were most susceptible to fosfomycin and imipenem (p = 0.01). The organisms were resistant to ceftazidime (p = 0.01).

Conclusion

In Pakistan, most patients with resistance present with mild symptoms instead of severe clinical manifestations. Therefore, there is a need to reduce the over-prescription of antibiotics for urinary tract infections, especially in cases when other non-antimicrobial agents can be used.

## Introduction

Urinary tract infection is one of the common diseases present in the general population and affects a wide range of age groups [1,2]. The infections can involve both the upper and lower urinary tract, and therefore, patients present with a variety of symptoms. Cystitis, a lower urinary tract infection, manifests as dysuria, urgency, frequency, and sometimes, suprapubic tenderness. However, similar features are seen in upper urinary tract infections. Therefore, it becomes difficult to distinguish between the two unless there are other symptoms, such as a history of recurrence, anatomical malformations, fever, and systemic signs. In these few cases, the urinary tract infections are either complicated or uncomplicated [3]. There are many management options for urinary tract infections. In cases of acute and uncomplicated cystitis, fosfomycin-trometamol, nitrofurantoin, nitroxoline, pivmecillinam, and trimethoprim are first-line agents [4]. In cases of acute uncomplicated pyelonephritis, the drug of choice is fluoroquinolone [5]. Parenteral agents are indicated in cases of complicated urinary tract infections [6]. The decision regarding the drug of choice as empirical treatment becomes difficult in the current era of antimicrobial resistance [7,8].

The objective of the study is to determine the pattern of bacterial agents responsible for urinary tract infections in a tertiary care hospital and to assess their antibiotic susceptibility and resistance pattern.

## Materials and methods

This is a prospective cross-sectional study that was conducted at Benazir Bhutto Hospital, Rawalpindi over a period of eight months. The sample size was calculated to be 440 urine samples using Open Source Epidemiologic Statistics for Public Health with a confidence interval of 95%, a design effect of 1 for a random sample, and an anticipated frequency of 50% [9]. The expected population was estimated using the average monthly outpatient workload. The sampling technique was non-probability consecutive sampling. Informed consent was sought from all the participants. The study setting was the outpatient department of the hospital. The inclusion criterion was patients presenting with symptoms of urinary tract infection. The exclusion criteria of the study were patients who had refused to participate, patients who had been admitted to the hospital for management, and patients who had presented with a prediagnosed condition of the urinary tract. The urine samples of these patients were then taken to confirm the diagnosis. The culture and sensitivity results for the specimens were recorded on a proforma. The data were analyzed using IBM Statistical Package for Social Sciences (SPSS) version 22 (IBM Corp., Armonk, NY). Descriptive statistics were used to describe the data. Chi-square test was applied to determine the significance of the difference between gender and microorganisms as well as microorganism and antimicrobial sensitivity. P-value of less than 0.05 was considered significant.

## Results

There were a total of 440 samples collected from patients. Out of 440 samples, 144 culture-positive samples had been obtained from male patients and 296 culture-positive samples had been obtained from female patients. The most common organism obtained from the urine culture was *Escherichia coli* (*E. coli*) (75%). The organisms are further detailed in Table 1.

**Table 1:** Microorganism yield in both genders

Microorganism	Gender	
	Frequency of males (%)	Frequency of females (%)
Escherichia coli	107 (24)	223 (51)
Klebsiella	13 (3)	32 (7)
Enterococcus spp.	8 (2)	16 (4)
Staphylococcus aureus	5 (1)	12 (3)
Pseudomonas aeruginosa	7 (2)	5 (1)
Acinetobacter	2 (0)	4 (1)
Enterobacter	1 (0)	4 (1)
Proteus mirabilis	1 (0)	0 (0)

The difference in the spectrum of microorganisms between the genders was not significant. The sensitivity of organisms to various antimicrobial agents was also noted and shown in Table 2. Chi-square test was applied to determine if the difference was significant. P-value of less than 0.05 was considered significant.

**Table 2:** Sensitivity of organisms to various antimicrobial agents p < 0.05 was significant

Antimicrobial agent	Frequency of sensitive cultures (%)	Frequency of resistant cultures (%)	p-value
Amoxicillin and clavulanic acid	120 (27)	320 (73)	0.11
Aztreonam	184 (42)	256 (58)	0.38
Amikacin	435 (99)	5 (1)	0.99
Gentamicin	230 (52)	210 (48)	0.12
Cephalexin	275 (63)	165 (37)	0.22
Clarithromycin	153 (35)	287 (65)	0.61
Ceftazidime	93 (21)	347 (79)	0.01
Ceftriaxone	121 (28)	319 (72)	0.85
Ciprofloxacin	153 (35)	287 (65)	0.21
Nitrofurantoin	406 (92)	34 (8)	0.75
Fosfomycin	426 (97)	14 (3)	0.01
Imipenem	376 (85)	64 (15)	0.01
Meropenem	400 (91)	40 (9)	0.07

The urine cultures from female patients had a wide spectrum of sensitivity (38.86%) as opposed to samples that had been obtained from male patients (20.91%). *Escherichia coli* was found to be sensitive to piperacillin and tazobactam (75.27%), cefoperazone and sulbactam (72.50%), vancomycin (4.54%), azithromycin (0.22%), teicoplanin (0.22%), and tigecycline (1.36%).

## Discussion

The results of the study showed a 70:30 ratio between female and male participants in terms of culture-positive urine samples. This finding confirms the fact that females are at a higher risk of developing urinary tract infections [10,11]. This can be attributed to the fact that females have a short urethra [12]. Changes in estrogen levels are also responsible. Lower levels of estrogen cause the normal vaginal flora made up of *Lactobacillus* to decrease [13].

The most common organism was *E. coli*. Uropathogenic species of the organism possess various characteristics that provide them with an advantage over the normal immunity mechanisms. Fimbriae and pili increase the attachment of *E. coli* to host cells, whereas biofilm increases the adherence to the uroepithelium [14]. The presence of the biofilms also provides resistance against normal antimicrobial agents, which explains the high proportion of resistant cultures in our study.

The results showed that the majority of the cultures were sensitive to nitrofurantoin (p = 0.75) and fosfomycin (p = 0.01). The patients, therefore, might have presented with uncomplicated urinary tract infections and mild symptoms. This finding also implies that the urinary tract infection was caused by extended-spectrum β-lactamase *E. coli*. Nitrofurantoin and fosfomycin are recommended for first-line therapy in such subcategories because of the sensitivity of *E. coli* to both these agents [15].

This is a serious issue because extended-spectrum β-lactamase *E. coli* has a high sensitivity to ceftazidime and meropenem [16]. However, in our study, the majority of the cultures were found to be resistant to the agents. Furthermore, the sensitivity of the organism was high in the case of imipenem. This was a peculiar finding because there was a high rate of resistance for meropenem (91%). Both these agents are considered highly efficacious for treating urinary tract infections but imipenem is emerging as a single agent therapy because of its pharmacodynamics characteristics [17].

A peculiar finding was the presence of significant resistance in cultures obtained from male participants, despite the lower yield of *E. coli* when compared to females. This could be related to the ratio of participants. This could also be related to the aging process in males. With increasing age, men tend to have voiding difficulties. These difficulties can generate turbulent retrograde urine flow leading to ascending infections. The prostate might be ultimately involved and it becomes a site for multiplication of resistant bacteria [18]. The extended-spectrum of resistance did not explain the sensitivity of *E. coli* to lactamase inhibitor combinations in drug therapy as well as macrolides and other agents because these species are more than likely to be resistant to the aforementioned drugs.

There are a few limitations to the study. The study was done at one centre. The study did not aim at dividing patients into appropriate age groups for risk stratification. The symptoms of the participants were not explored intensively. Antimicrobial agents within the same subgroup had not been compared.

## Conclusions

Increasing antimicrobial resistance is a big concern for healthcare facilities worldwide. In Pakistan, the problem raises more concern, because most patients with resistance present with mild symptoms instead of severe clinical manifestations. There is a need to address the over-prescription of antimicrobial agents to combat this problem.
